# Patient compliance with NHS 111 advice: Analysis of adult call and ED attendance data 2013–2017

**DOI:** 10.1371/journal.pone.0251362

**Published:** 2021-05-10

**Authors:** Jen Lewis, Tony Stone, Rebecca Simpson, Richard Jacques, Colin O’Keeffe, Susan Croft, Suzanne Mason

**Affiliations:** 1 School of Health and Related Research (ScHARR), University of Sheffield, Sheffield, United Kingdom; 2 Sheffield Teaching Hospitals NHS Foundation Trust, Sheffield, United Kingdom; Technion - Israel Institute of Technology, ISRAEL

## Abstract

The NHS 111 telephone advice and triage service is a vital part of the management of urgent and emergency care (UEC) services in England. Demand for NHS 111 advice has increased since its introduction in 2013, and the service is of particular importance in light of the current pandemic and resulting increased demand for emergency care. Currently, little is known about the effectiveness of NHS 111 in terms of the appropriateness of the advice given, or about the compliance of patients with that advice. We aimed to address this issue by analysing a large linked routine dataset of all NHS 111 calls (n = 3,631,069) and subsequent emergency department (ED) attendances made in the Yorkshire & Humber region from March 2013-March 2017. We found that many patients do not comply with advice, with 11% (n = 289,748) of patients attending ED when they are advised to self-care or seek primary care. We also found that a considerable number of these patients are further classed as urgent (88%, n = 255,931) and a substantial minority (37%, 106,207) are subsequently admitted to hospital. Further, many patients who are sent an ambulance or told to attend ED are classed as non-urgent upon attending ED (9%, n = 42,372). This research suggests that the level at which NHS 111 is currently triaging results in many hundreds of thousands of mis-triaged cases annually. Additionally, patients frequently do not comply with the advice they receive. This has implications for understanding the accuracy and efficiency of triaging systems.

## Introduction

The NHS 111 telephone advice and triage service was set up in 2013 to improve access to urgent and emergency care (UEC) for patients in England and direct them to the most appropriate level of care for their health needs. The NHS Long Term Plan is committed to NHS 111 as part of an integrated urgent care approach to reduce pressure on hospital services [1].

A number of studies have been conducted examining the impact of NHS 111 in reducing demand on urgent care systems. Whilst initially, patient satisfaction with the service was high and patients tended to comply with the advice given [2], it is unclear whether this has changed as the service has evolved and use has become more widespread with increased demand. Studies have suggested that the service has struggled to meet targets, establish a positive impact on the wider urgent care system or demonstrate cost-effectiveness [3], and frequent non-compliance with advice has been documented [4]. Some studies have implied that NHS 111 may lead to increased demand and/or use of some UEC services [5, 6], though it is not clear what may be driving this trend. However, other evidence suggests NHS 111 may prevent a considerable number of unnecessary emergency department (ED) visits [7], for example by directing them to primary care [6], suggesting the overall impact of NHS 111 on demand for emergency services is more nuanced.

NHS 111 is staffed primarily with ‘call handlers’–advisers without a specialist clinical background. Though some clinical advisers are also available to support, particularly with more challenging cases, this staffing decision has received particular criticism, and is suggested to be the cause of increased pressure on emergency services. Studies suggest that those with a clinical background, such as GPs and nurses, tend to be markedly less risk averse in their recommendations to callers [8, 9], and paramedics attending patients referred to the ambulance service by NHS 111 have expressed frustration at overly cautious triaging by non-clinical call handlers [10]. Evidence also suggests that callers are more likely to make an avoidable ED attendance if they receive advice only from a call handler and not from a clinical adviser [4], which may compound already cautious triaging.

NHS 111 is a widely-used service which answered calls from almost 15 million people in 2018/19 [7]. At least 17,328,198 calls were offered in 2019 including abandoned and unanswered calls [11]. However, there remains a lack of robust evidence on the impact of NHS 111 on subsequent use of hospital services. This is particularly lacking in studies using linked data to follow individual patient episodes of care to understand the extent to which NHS 111 recommendations are appropriate, and the extent to which they are followed by patients.

During the recent COVID-19 pandemic, NHS 111 services were relied upon to triage many concerns from the public about managing suspected symptoms and health implications, and there is an increasing focus on the role of NHS 111 in managing demand for emergency healthcare in the context of COVID-19 [12]. Call volume during the spring of 2020 rose dramatically, but many calls went unanswered, and whether callers tended to follow advice remains unclear [13]. There is now much scrutiny over how to deliver robust healthcare in pandemic situations, but research to date has been unclear on how the advice given by NHS 111 is followed by the public.

The aim of this analysis was to measure attendance at ED (and subsequent hospital admissions) up to two days after an NHS 111 call, and whether this is in line with the recommendations given by NHS 111 staff.

## Materials and methods

### Data

We used a dataset extracted from the “Connected Health Cities: Data linkage of urgent care data” study (known as the “CUREd research database”) [14]. The CUREd database has approval from a National Health Service (NHS) Research and Ethics Committee, overseen by the NHS Health Research Authority’s Research Ethics Service (18/YH/0234), and from the NHS Health Research Authority (HRA), directly (18/CAG/0126, previously 17/CAG/0024), to receive health and social care data without patient consent for patients of emergency and urgent care services in Yorkshire and Humber.

The CUREd dataset includes routinely collected healthcare data for all NHS 111 calls in the Yorkshire & Humber region of the UK from the beginning of April 2013 to the end of March 2017. It also includes data concerning ED attendances and all emergency hospital admissions from 13 NHS Acute Trusts across Yorkshire & Humber. The data used in the study comprised de-identified data extracted from routinely collected information on every NHS 111 call made in the region between the specified dates. Additionally, it included any attendance at a type 1 ED (a consultant-led 24 hour service with full resuscitation facilities and designated accommodation for the reception of ED patients) linked to the corresponding patient within 48 hours of the NHS 111 call, and any subsequent hospital admission made up to one night after an ED attendance, indicated by an admitted patient care (APC) record for that patient. Multiple NHS 111 calls from a single patient are included, however, for the present analysis only the final call within 48 hours before an ED attendance was linked to that attendance, to avoid multiple representations of attendances. The data included only those patients aged 16 or over at the time of the NHS 111 call.

### Data linkage

ED attendances and hospital admissions were traced following an NHS 111 call by the linkage of the routine datasets listed above. The aim was to identify all records amongst the datasets that correspond to the same individual. This was achieved using the following method:

1. Assign each distinct (valid NHS Number, valid date of birth [DOB]) pair a new CUREd ID.
2. Attempt to link records with a valid NHS Numbers but no valid DOB to a CUREd ID based on approximate birth year (calculated from activity date and age at activity).
3. Attempt to link remaining records to an assigned CUREd ID by Provider code, Provider Patient ID and DOB matches (provided this only matches one CUREd ID).
4. Attempt to link remaining records to an assigned CUREd ID by First name, Last name, Sex, DOB and postcode matches (provided this only matches one CUREd ID).
5. Attempt to link remaining records to an assigned CUREd ID by Sex, DOB and postcode matches (provided this only matches one CUREd ID).
6. Cluster records by agreement on any of the following patterns:
  - Provider code, Provider Patient ID and DOB matches
  - First name, Last name, Sex, DOB and postcode
  - Sex, DOB and postcode matches and assign each distinct cluster to a new CUREd ID.
7. Assign each remaining record to its own CUREd ID.

### Analysis

For this exploratory investigation, all analyses were descriptive in nature. For each NHS 111 call record in the dataset pertaining to an adult patient (age 16+), we determined whether an ED attendance was made by that patient within 48 hours of the call, and if so, whether this was followed by an emergency hospital admission. This examination was broken down by the category of advice given to each caller. These categories are known as ‘dispositions’ and include:

1. Ambulance dispatch. The NHS 111 call handler transfers the call to 999 for an ambulance response. Note that not all ambulance dispatches will result in further transportation to ED, with this being deemed unnecessary by paramedics, or patients being conveyed directly to ward bypassing ED.
2. Attend ED. The caller is advised to attend ED within a given time frame by their own means.
3. Primary or community care. The caller is advised to seek primary or community care, for example a GP, dental practitioner or community pharmacy, within a given time frame.
4. Self care. The caller is not advised to seek additional care services. This also includes incidences where the caller abandoned the call without receiving a recommendation.
5. Other service. The caller is advised to seek care from a service not covered by the above categories. Services included will depend on their local service configurations but may include, for example, mental health services, a district nurse, or midwife.

We examined the proportion of callers in each disposition, the proportion of callers attending ED within 48 hours of the NHS 111 call, and the proportion of ED attendees either classed as non-urgent or experiencing a subsequent hospital admission. Non-urgent cases were defined as attendances at a type 1 ED, as a first attendance, but who were subsequently identified as not receiving investigations, treatments or referral that required the facilities of a type 1 ED [15]. Calls were split by various categories and combinations thereof to explore possible differences in patient flow. These categories included:

- Disposition
  ○ Ambulance dispatch, ED, Primary or community care, Self-care, Other service
  ○ Detailed disposition code including recommended time to access care
- Age category
  ○ 16–44, 45–74, 75+
- Clinical adviser handled call
  ○ Yes, no
- Year of call
  ○ 2013–2017

Outcomes considered for each category included:

- Percentage of callers attending ED within 4, 24, or 48 hours of the NHS 111 call;
- Time taken to attend ED;
- Percentage of ED attendances classed as non-urgent;
- Percentage of ED attendances admitted.

## Results

In total, 3,631,069 calls were made to NHS 111 regarding adult patients. There was a small amount of missing data (n = 16,154, 0.4%). Of 781,561 patients attending ED from all dispositions, 78,056 were defined as non-urgent (10.0%), and 301,677 (38.6%) were subsequently admitted. The remaining 51.4% of attendees were discharged after receiving investigations, treatments or referral requiring ED facilities.

**Fig 1:**
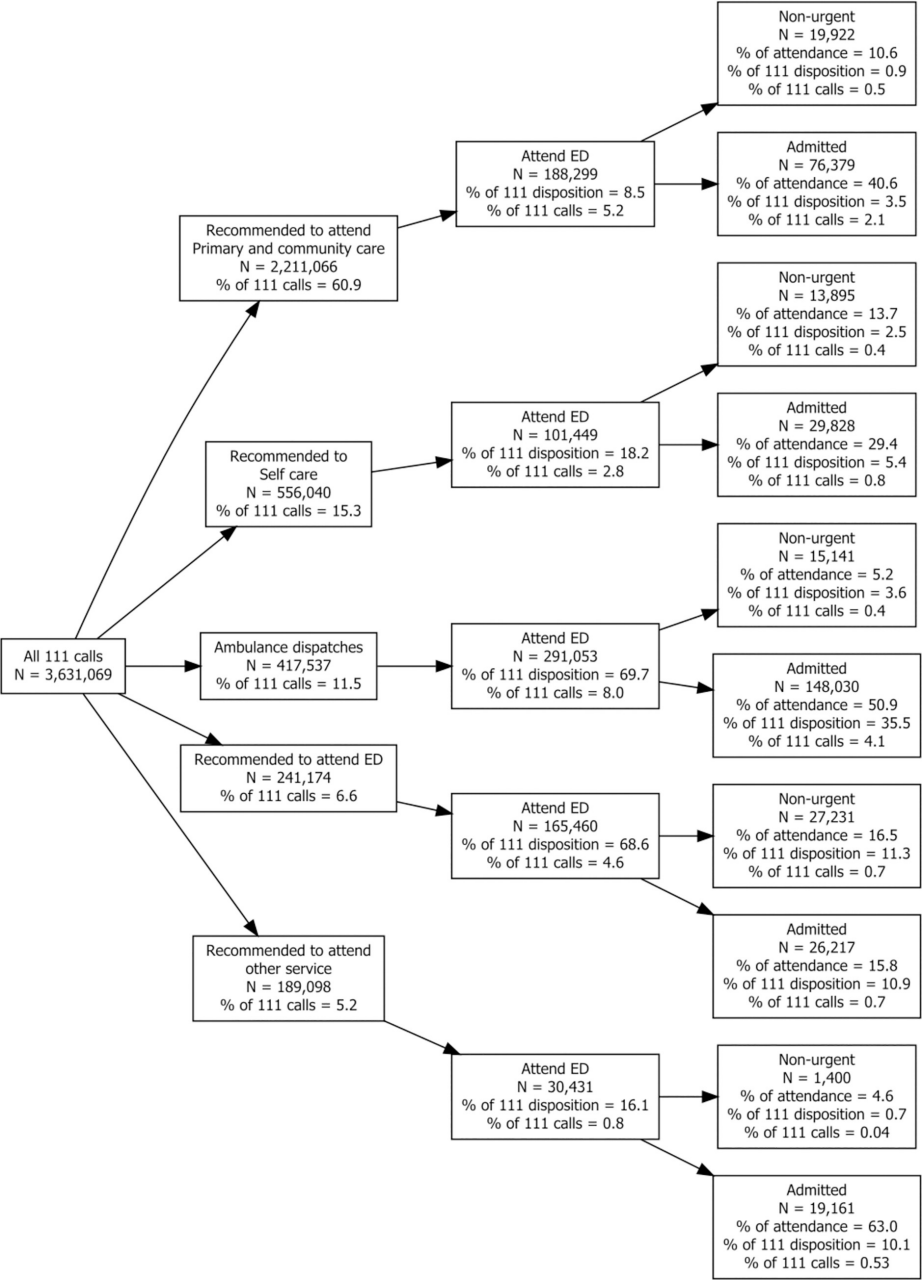
Patient flow for all adult callers to NHS 111, split by disposition. ‘Attend ED’ refers to those attending within 48 hours of the NHS 111 call. Not shown: 16,154 calls with missing disposition data (0.4%).

The data revealed that the majority of adults callers are advised to self-care, seek community care (e.g. pharmacy) or seek primary care advice (n = 2,767,106, 76.2%). Where primary care is advised, this may range from advice to seek help within 1 hour to up to 3 days. An ambulance was dispatched for 11.5% of calls (n = 417,537). Advice to attend ED was given for 6.6% of calls (n = 241,174). However, a considerable percentage of patients from all dispositions, including low-acuity dispositions, subsequently attended ED (Table 1): in total we found that 320,179 patients attended ED despite being advised to self-care, seek primary care or attend another service (10.8%), and 125,368 (39.2%) of these patients were subsequently admitted. Of patients receiving a high-acuity disposition, 456,513 (69%) attended ED, but approximately the same proportion of these patients were admitted as those given low-acuity dispositions (174,247; 38.2%). Fig 1 displays the flow of patients from NHS 111 call to post-ED attendance split by disposition.

**Table 1:** ED attendance details for NHS 111 callers broken down by various categories.

	NHS 111 callers	NHS 111 callers attending ED		
	N attending ED by category (%)	Median hours to attend (IQR)	N arriving within 4 hours (%)	N arriving within 24 hours (%)
**Disposition**				
Primary/community care	188,299 (8.5)	2.84 (1.6, 7.0)	116,844 (62.1)	171,974 (91.3)
Self-care	101,449 (18.2)	1.05 (0.7, 1.9)	88,680 (87.4)	98,012 (96.6)
Ambulance dispatched	291,053 (69.7)	1.41 (1.1, 1.9)	282,704 (97.1)	289,755 (99.6)
Attend ED	165,460 (68.6)	0.95 (0.7, 1.5)	155,837 (94.2)	163,991 (99.1)
Attend other	30,431 (16.1)	2.33 (1.5, 4.7)	21,562 (70.9)	28,614 (94.0)
**Age category**				
age16-44	371,750 (19.2)	1.28 (0.8, 2.2)	320,485 (86.2)	359,639 (96.7)
age45-74	239,833 (23.3)	1.44 (1.0, 2.3)	207,968 (86.7)	233,044 (97.2)
age75+	169,978 (25.4)	1.76 (1.3, 2.9)	141,750 (83.4)	164,489 (96.8)
**Call year**				
2013	104,147 (20.7)	1.32 (0.9, 2.2)	90,017 (86.4)	100,673 (96.7)
2014	181,041 (20.7)	1.40 (1.0, 2.3)	155,685 (86.0)	175,226 (96.8)
2015	206,148 (21.3)	1.48 (1.0, 2.5)	176,550 (85.6)	199,682 (96.9)
2016	230,664 (22.5)	1.48 (1.0, 2.5)	197,662 (85.7)	223,807 (97.0)
2017	59,561 (22.8)	1.52 (1.0, 2.6)	50,289 (84.4)	57,784 (97.0)
**Adviser**				
Call handler	621,851 (21.7)	1.36 (0.9, 2.2)	540,624 (86.9)	603,496 (97.0)
Clinical adviser	159,710 (20.9)	1.85 (1.2, 3.2)	129,579 (81.1)	153,676 (96.2)

We will examine each of the three main disposition groups (primary/self-care, ambulance response, ED attendance) and describe their journey following their NHS 111 contact.

### Call outcomes by NHS 111 disposition category

In this section we further examine trends for those patients given a disposal code for self or primary care, ambulance dispatch and recommendation to attend ED. We do not explore those provided an ‘Other service’ disposal code, since this represents a heterogeneous group for whom generalisations are unlikely to be informative.

#### Low acuity dispositions: Self-care & primary care

**Fig 2:**
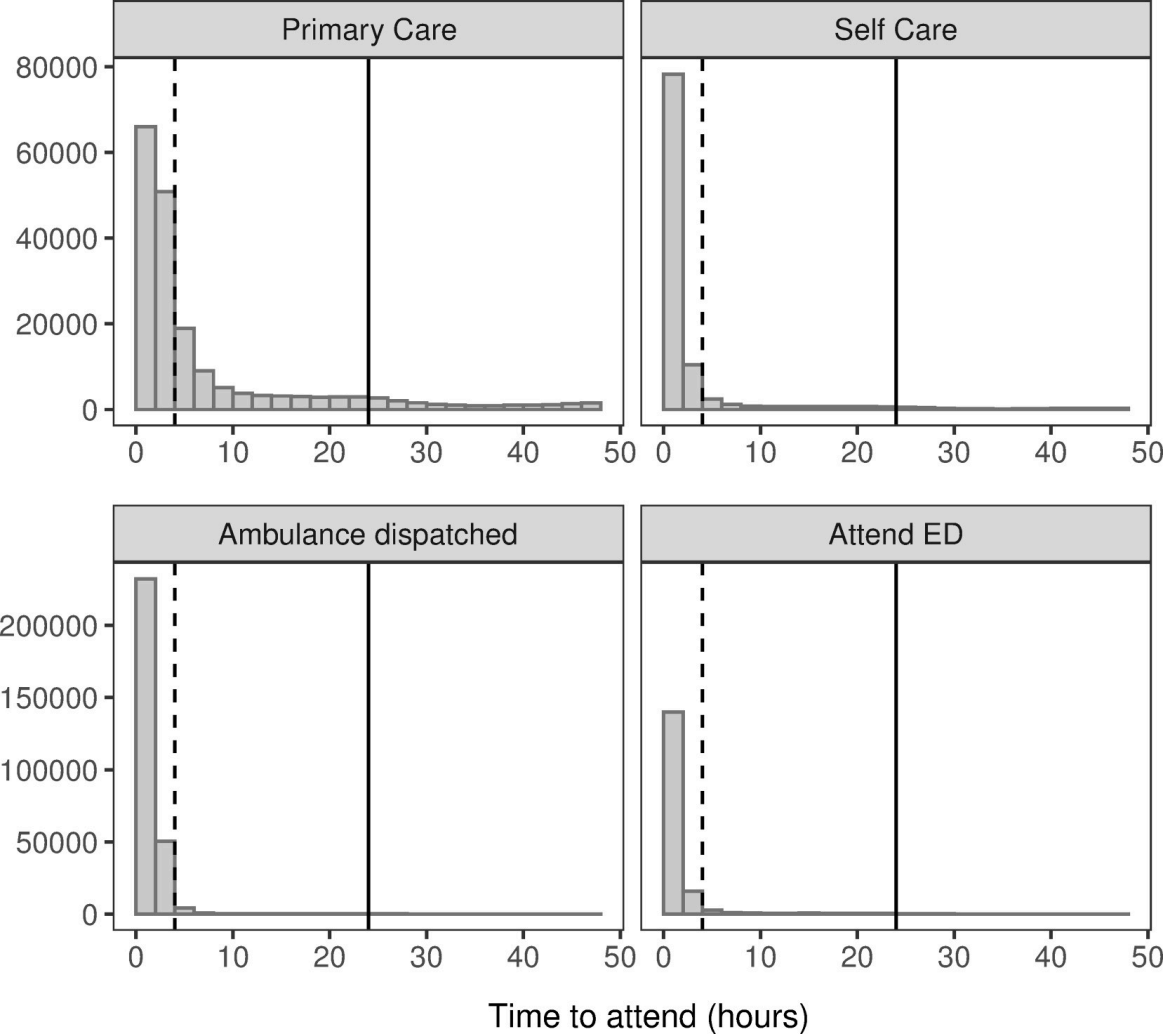
Distribution of hours taken to attend ED for those advised to different dispositions. Solid vertical lines denote 24 hours from the call; dashed lines denote 4 hours.

Using our linked dataset we identified that of those advised to self-care or contact primary / community care, 10.5% (n = 289,748) attended the Emergency Department (ED) within 48 hours of the NHS 111 call. A greater percentage of those patients advised to self-care than to access primary care decided to attend the ED (18.2% vs 8.5%). When we analysed the time of attendance at ED, we found that the majority of patients attending ED do so within 4h of their NHS 111 call. This is especially the case for those adults advised to self-care, compared to those advised to access primary care (87.4% vs 62.1% of callers). For adults advised to seek primary care, Fig 2 shows a longer and heavier tail to the distribution of attendance time.

Of those callers initially advised to seek primary or community care, or to self-care, but who subsequently attended ED, 36.7% (n = 106,207) were then admitted to hospital (equating to 3.8% of all callers in this disposition). We found within this disposition group that 11.7% of attendances at the ED were non-urgent (n = 33,817).

We further examined final disposition codes provided by NHS 111 for each call. We found that those given a primary care disposition (either as face to face, or telephone contact) recommended within 1–2 hours had a higher likelihood of resorting to an ED attendance than those requiring primary care over a longer time period (Table 2). They also attended faster: the average time of attendance at ED for shorter-timescale patients was around 2.5–3 hours following the NHS 111 call, whereas those receiving a longer-timescale disposition (12 hours or more) was around 3.5–6 hours.

**Table 2:** Time to attend ED for those advised to contact primary care within different time intervals.

	NHS 111 callers advised to access PC	NHS 111 callers attending ED after advice to access PC		
Recommended time to access PC	Total attending ED by category (%)	Median hours to attend (IQR)	N arriving within 4 hours (%)	N arriving within 24 hours (%)
1 hour	30,769 (15.1)	2.70 (1.7, 5.3)	20,478 (66.6)	28,851 (93.8)
2 hours	80,827 (13.2)	2.66 (1.6, 5.8)	52,813 (65.3)	74,821 (92.6)
6 hours	47,935 (8.3)	2.96 (1.6, 7.8)	28,773 (60.0)	43,483 (90.7)
12 hours	11,778 (4.0)	3.47 (1.5, 12.7)	6,306 (53.5)	10,343 (87.8)
24 hours	14,724 (3.4)	3.85 (1.5, 15.7)	7,491 (50.9)	12,647 (85.9)
Other	2,266 (3.0)	5.70 (1.7, 21.2)	983 (43.4)	1,829 (80.7)

#### Ambulance dispatches

We were able to identify patients who had an ambulance dispatched for them by NHS 111 staff. Fig 1 shows that 11.5% (n = 417,537) of all calls to the NHS 111 service resulted in an ambulance dispatch. Many of these patients were transported to ED (n = 291,053, 69.7%), though a considerable number (~30%) were not. Not surprisingly, the majority of patients receiving an ambulance response arrived quickly at the ED, most doing so within 2 hours (Fig 2). Of those arriving at ED, 50.9% of patients were admitted to hospital (n = 148,030). Only 5.2% (n = 15,141) patients met our definition of a non-urgent attendance when arriving by this route.

#### Emergency department attendance

Linked data was able to identify those advised to attend ED by the NHS 111 service, who then actually attended ED. We found that, of the 6.6% of calls advised to attend (n = 241,174), 68.6% actually did so (n = 165,460), and the vast majority attended within 4 hours (Fig 2, Table 1). However, a substantial proportion of those callers advised to attend ED did not do so (~30%). Once attending ED we found that 15.8% of those attending were admitted (n = 26,217), while 16.5% of those attending were classed as non-urgent (n = 27,231).

#### Change over time

Overall there was a slight increase in the proportion of NHS 111 calls that were followed by an ED attendance in the years studied (Table 1), in line with previous reports [6]. However, in general, both the percentages of patients attending ED from each disposition, and the median time taken to attend, changed little over time (S1 Fig).

### Examination of subgroups

#### Age group

We examined details for patients of different ages to identify any systematic differences in these groups. There was little apparent difference in the average time to attend, and the percentage of callers attending ED from different age groups when ignoring disposition (S2 Fig, Table 1). In all age brackets, the majority of those attending ED did so within 4 hours, and almost all within 24 hours.

**Fig 3:**
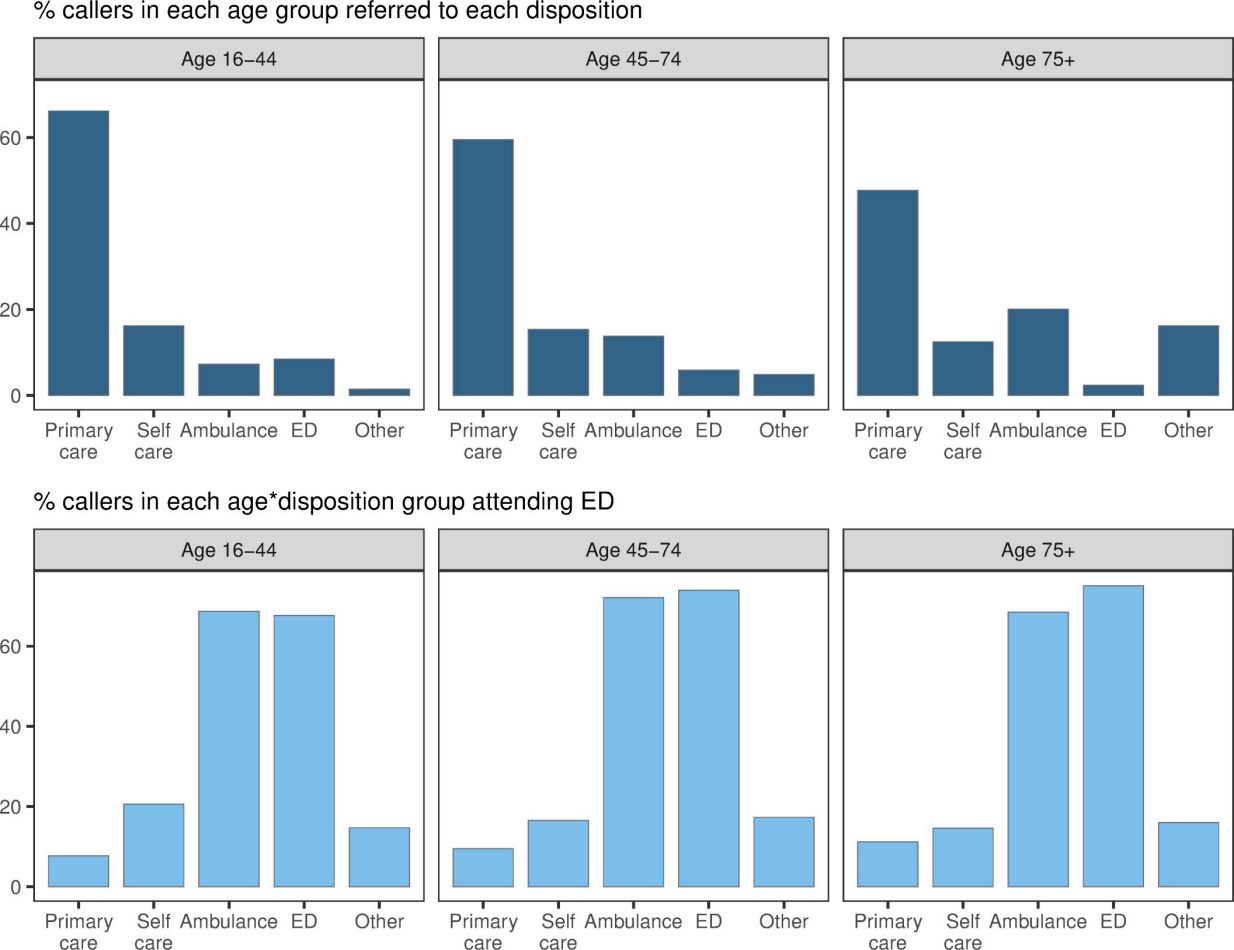
Proportion of NHS 111 callers given each disposition and attending ED, split by age. Top: percent of NHS 111 callers in each age group receiving each disposition; e.g., 66.2% of 16–44 year-olds were advised to contact primary care. Bottom: percent of NHS 111 callers in each age-disposition group who attended ED; e.g. 7.7% of 16–44 year-olds who were advised to contact primary care attended ED.

When age groups are further broken down by disposition, some patterns of interest are revealed (Fig 3). There were fewer primary care and self-care disposals among older patients. In contrast, more patients in the oldest group were sent an ambulance or directed to other healthcare services. Few older patients were advised to attend ED–likely due to increased barriers to transport or greater concern regarding older patient welfare. While there were differences in the profile of dispositions, similar percentages of patients attend ED from each disposition, regardless of their age bracket. In total, around 21% of patients in the youngest age bracket attend ED, compared to around 28% of patients in the oldest age bracket.

**Fig 4:**
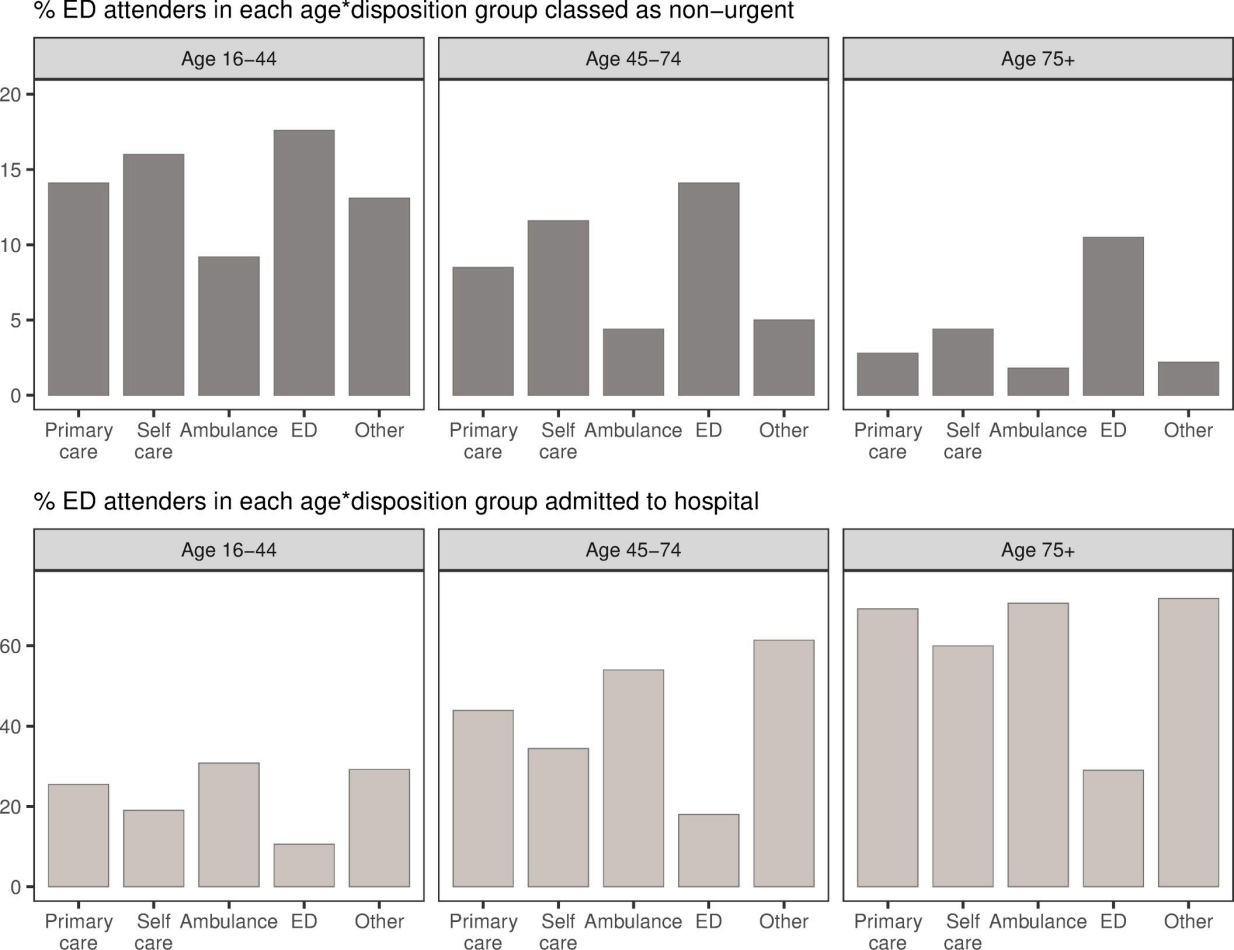
Proportion of ED attendees from each disposition considered non-urgent, and those admitted, by age. Top: percent of NHS 111 callers in each age-disposition group who attended ED, who were classed as non-urgent; e.g., 14.1% of callers aged 16–44, who received a primary care disposition but attended ED, were classed as non-urgent. Bottom: percent of NHS 111 callers in each age-disposition group who attended ED, who were subsequently admitted; e.g., 25.5% of callers aged 16–44, who received a primary care disposition but attended ED, were admitted. Note that relative numbers of patients in these disposition groups are markedly different (see Fig 3).

We found a notable difference in the relative percentages of ED attendees that were subsequently admitted to hospital in the difference age groups (Fig 4). Far higher proportions of older patients were admitted following an ED attendance than younger patients. This held even for low acuity dispositions; for example, nearly 60% of older patients who attended ED, who were originally advised to self-care, were admitted to hospital. This is in contrast to the same group in the youngest age bracket, of whom just over 20% were admitted. In all age groups, and across all dispositions, there were relatively few ED attendees who were classed as non-urgent. Indeed, in all but one subgroup (16–44, advised to attend ED), larger numbers of patients were subsequently admitted than were classed as non-urgent.

#### Clinical advisers

**Fig 5:**
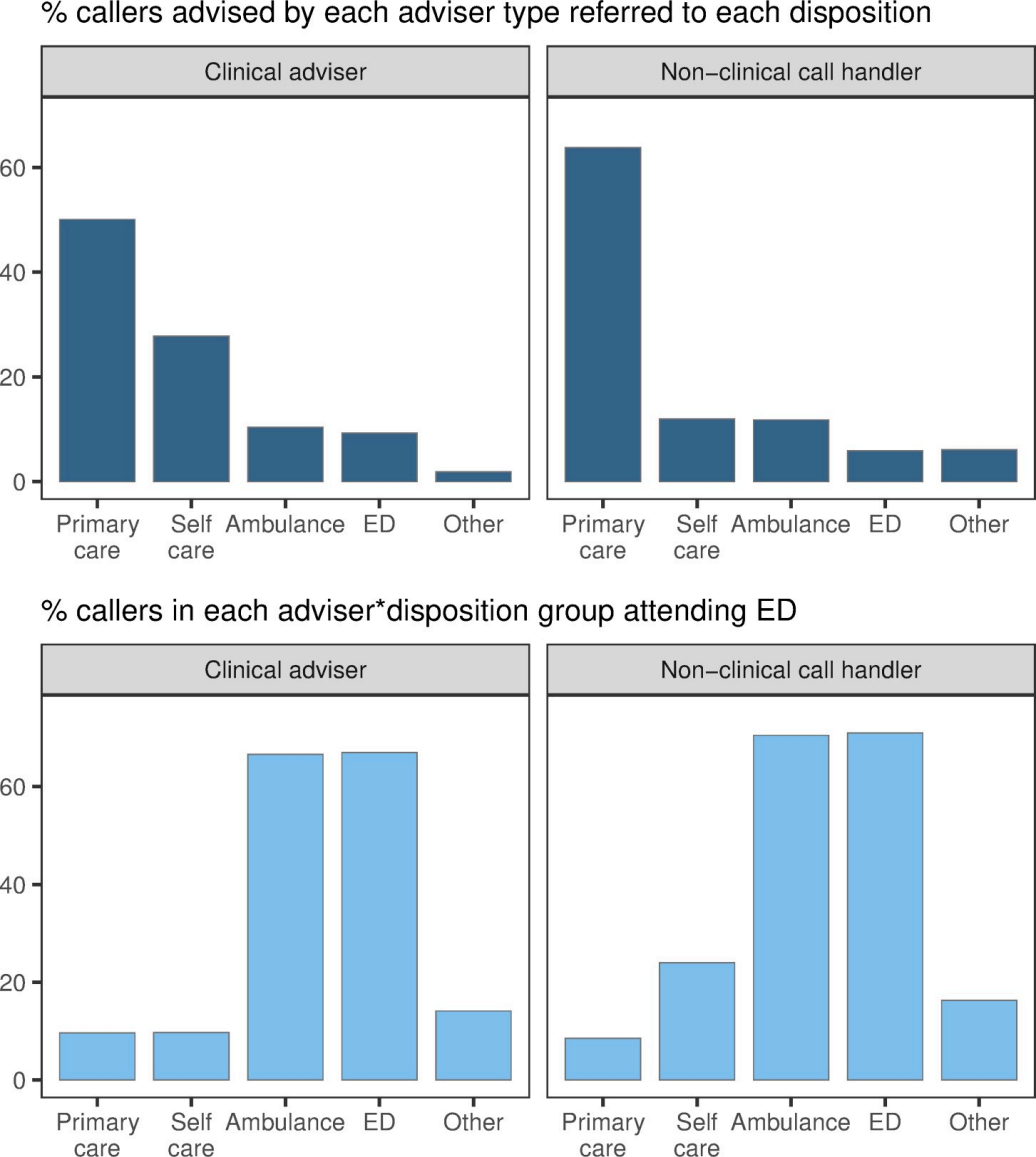
Proportion of callers given each disposition and attending ED, split by call handler. Top: percent of NHS 111 callers advised by each adviser type receiving each disposition; e.g., 50.1% of those advised by a clinical adviser received a primary care disposition. Bottom: percent of NHS 111 callers in each adviser-disposition group who attended ED; e.g. 9.6% of those advised by a clinical adviser who received a primary care disposition attended ED.

Finally, we explored the data for any differences in attendance behaviour depending on whether a clinical adviser handled the NHS 111 call or not. As with age groups, we found little difference overall on the average time to attend, or percentage of callers attending ED, based on the recommendation of a clinical adviser (S3 Fig, Table 1). Again, however, once broken down by disposition, some differences were revealed between calls taken by clinical advisers and non-clinical call handlers (Fig 5; although it is important to note that clinical advisers may be more likely to deal with borderline or complex cases–see discussion). Clinical advisers tended to direct fewer patients to primary/community care or other care services, and more to care for themselves. This may reflect a greater confidence in determining minor health issues among this group.

**Fig 6:**
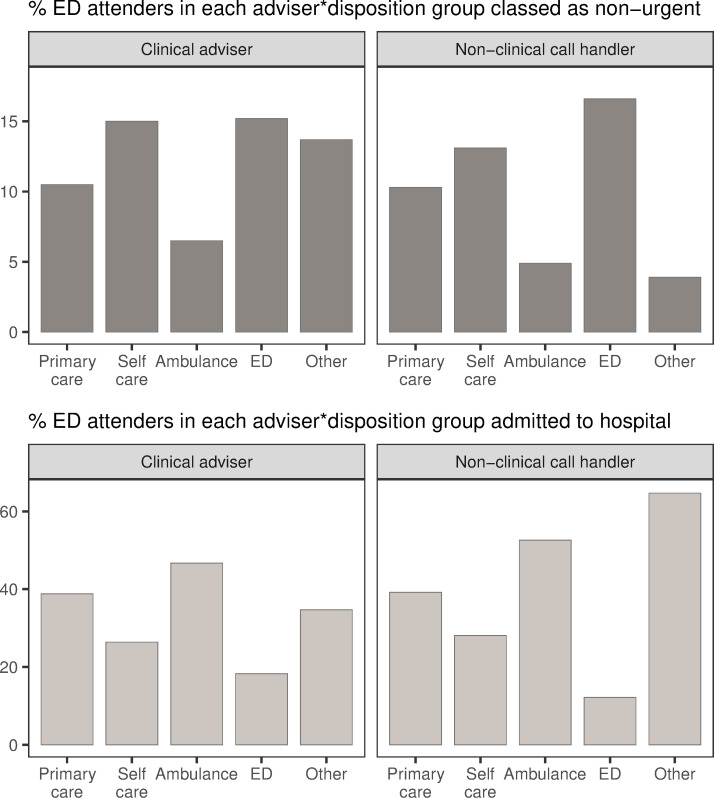
Proportion of ED attendees from each disposition considered non-urgent, and those admitted, by call handler. Top: percent of NHS 111 callers in each adviser-disposition group who attended ED, who were classed as non-urgent; e.g., 10.5% of callers who were advised by a clinical adviser and received a primary care disposition but attended ED, were classed as non-urgent. Bottom: percent of NHS 111 callers in each adviser-disposition group who attended ED, who were subsequently admitted; e.g., 38.8% of callers who were advised by a clinical adviser and received a primary care disposition but attended ED, were admitted. Note that absolute numbers of patients in these disposition groups are markedly different (see Fig 5).

Generally there were few differences in the proportions of patients attending ED, or classed as non-urgent, from each disposition regardless of the type of adviser. However, callers seem slightly more likely to follow advice to self-care given by a clinical adviser, which may reflect more faith in the adviser’s decision (Fig 5). In all but one group, more patients were admitted to hospital than were classed as non-urgent, and this difference was often marked. The one exception to this is, as before, were patients who were originally advised to attend ED; specifically those who were advised by a non-clinical call handler. The main difference between clinical advisers and non-clinical call handlers is the proportion of patients originally directed to other services. In this group, far more ED attendees who were advised by a non-clinical call handler were subsequently admitted to hospital (Fig 6). This may reflect a better understanding held by clinical advisers of other services available and the appropriate circumstances under which to direct patients to those services. Note, however, there are relatively few absolute numbers of patients in this category.

## Discussion

Our research is consistent with existing work showing a high number of avoidable ED attendances following an NHS 111 call [4], and an increasing number of ED attendances over time [6]. Additionally, our finding of around 10.8% non-compliance with advice by patients given a low-acuity disposition suggests a national figure of around 2.5 million unadvised ED attendances annually following an NHS 111 call, reflecting a large amount of resource usage, while performance against the four-hour ED waiting time standard continues to decline [16]. This raises the question of whether the current NHS 111 triaging systems are performing sufficiently accurately and confidently to ensure efficient use of NHS resources.

In light of evidence suggesting increasing dissatisfaction with GP services over the years covered in the present study, often due to a lack of staff or long waiting times [17], our findings may also support previous research suggesting that where there is less primary care availability, more avoidable ED attendances are made [18, 19]. In the present study, longer attendance times in the group advised to seek primary care (Fig 2) suggest that they may have first tried to make contact with primary care. To what extent this group comprises patients who have been further referred to emergency services by primary healthcare professionals is unclear, but Table 2 suggests that there may be insufficient services available for those patients who are advised to seek primary care within 1–2 hours. This is supported by evidence that many non-urgent patients who attend ED do so because a GP is unavailable, because the patient would be seen more quickly at ED, or because ED is easier to access than other services [20], although we note that patients advised to seek primary care within 1–2 hours may tend to have more serious complaints, and which may be more likely to worsen, possibly necessitating an ED visit.

Existing research has also suggested that the lack of clinical training leading to cautious triaging amongst NHS 111 call handlers may be responsible for high numbers of avoidable ED attendances and ambulance dispatch [8–10]. In support of this, many ambulance dispatches did not result in a conveyance to ED, and a large number patients did not attend ED despite being advised to do so (approximately 30%). In the case of ambulance dispatch, in some cases this may be due to conveyance straight to ward, telephone triage (‘hear and treat’), or discharge by the ambulance crew at the scene. However the large number of non-attendances at ED in both high-acuity dispositions suggests that patients themselves sometimes consider this recommendation to be overly cautious and choose not to continue with a transfer to 999. However, if this were exclusively due to cautious non-clinical call handlers, we might expect to see fewer non-urgent attendances following a call dealt with by a clinical adviser, whereas differences here were minimal. We note however it is likely that clinical advisers deal with more complex cases, and so the accuracy of decisions is probably not easily comparable between the two groups. Other possibilities include the presence of systemic miscommunication in NHS 111 calls, which previous research has suggested is common and can lead to diagnosis and assessment problems [21].

### Clinical implications

Following the recent pandemic, a ‘call-first’ model has been proposed by organisations such as the UK Royal College of Emergency Medicine [22], and currently being piloted in several regions across England [12, 23, 24]. This aims for NHS 111 to act as a non-compulsory ‘gatekeeper’ to emergency health services, and will encourage the public to call NHS 111 before attending other services, a model already practiced in some Scandinavian countries including Denmark [25]. The aim is for appropriate triaging of calls to manage suspected COVID-19 disease, but also to manage demand on systems more widely. Understanding the pathways of patients following calls to telephone helplines to ensure triaging is appropriate and effective is vital if future strategies include adopting the call-first model to manage demand and also risk in future pandemics. More broadly, understanding these pathways identifies areas in which the reasons for non-compliance and their interplay with the nature of NHS 111 advice must be further examined; this is crucial for future policy aimed at reducing non-urgent attendances and improving compliance.

During the COVID-19 crisis we have also seen primary care and hospital outpatients swiftly move from a face-to-face based service to telephone based in many instances, for patients both with and without suspected COVID-19 [26]. However there is a lack of evidence regarding safety, efficacy and impact of this approach. Understanding the role of NHS 111 within these new approaches is especially important for emergency care where access by the most marginalised in society will be vital–such as the elderly, those with chronic ill health and mental health problems.

### Further research

These findings raise additional questions for future investigation. It is particularly important to understand discrepancies in ED attendances and admissions: both why such a large proportion of patients given a low-acuity disposition are admitted to hospital when they do attend ED, and why relatively few patients directed to ED are subsequently admitted to hospital whereas a large proportion of these attendances are considered avoidable. In future work we will explore differences between those who are and are not subsequently admitted, and those who are classed as non-urgent to reveal insights about trends in these dispositions, at the level of detailed disposition codes with recommended time to access care where applicable. This will help to reveal insights regarding more detailed circumstances under which NHS 111 advice is ultimately not followed.

It is also of interest to explore the differences we found between age groups; specifically whether older patients are less likely to seek telephone help for less urgent healthcare issues, or whether call handlers and emergency care doctors are more cautious in their referrals and admissions of older patients, or that older patients’ lack of access to or provision of care at home leads to less urgent cases resulting in admission. The lack of differences between clinical and non-clinical advisers will also be investigated further in future work which will aim to understand differences in patient case-mix, treatment and diagnosis between those handled by clinical and non-clinical advisors.

### Strengths and limitations

This study represents an analysis of a large dataset covering 4 full years’ data, and thus provides a particularly comprehensive overview of patient pathways following a call to NHS 111. This is also the first study examining linked data to look at the post-call pathway in detail, including distributions of times to attend and details of the relative urgency of those who were and were not advised to attend ED. Our present data is also able to yield more detail regarding case-mix, symptoms and diagnoses, and multiple calls, which will be used to address further questions in future research.

However, unfortunately we currently do not have details regarding other services patients may access following a call, including GP services, so we are unable to draw conclusions about the possible role of onward referrals to ED at intervening points in the pathway; it is likely that in some cases, those advised to seek primary care were then further instructed by a GP to attend ED. In some cases patients may alternatively have been advised to either seek primary care or to self-care, but to attend ED if their condition worsened; unfortunately the data do not capture this level of detail at present. Similarly, we do not have patient level information regarding choices made, restricting the conclusions we are able to draw regarding the reasons for following or not following advice given by NHS 111 advisers.

## Conclusions

We found that around 10% of low acuity NHS 111 recommendations are not complied with. Of high acuity NHS 111 recommendations that are followed by patients, around 10% are found to be non-urgent and can be considered ‘mis-triages’. Our exploration suggests there may be areas in which the NHS 111 service may be systematically misclassifying the urgency of patient healthcare issues, or those in which other healthcare services may be underperforming (e.g., short notice and out-of-hours GP services, availability of home-based care for older patients) leading to overuse of ED. This potentially highlights the need for a more integrated approach to balancing primary and emergency healthcare service provision. However, these possibilities must be investigated and further elucidated before robust conclusions can be reached. Exploration of these issues may have implications for the understanding of the present efficiency of the NHS 111 service in reducing pressure on hospital services, and may point towards improvements that can be made in the future.

## Supporting information

S1 Fig Change over time in percentage of patients attending ED, and median time to attend, split by disposition and year of call. (Solid line = advised to attend ED; dotted line = ambulance dispatched; dashed line = directed to primary care; dot-dash line = advised to self-care).
(PNG)

S2 Fig Distribution of time taken to attend ED for patients across different age brackets. (PNG)

S3 Fig Distribution of hours taken to attend ED for patients advised by a call handler with or without clinical training. (PNG)
