# Correction: Patient compliance with NHS 111 advice: Analysis of adult call and ED attendance data 2013–2017

**DOI:** 10.1371/journal.pone.0307203

**Published:** 2024-07-10

**Authors:** Jen Lewis, Tony Stone, Rebecca Simpson, Richard Jacques, Colin O’Keeffe, Susan Croft, Suzanne Mason

The Data Availability statement for this paper is incorrect. The correct statement is: Retrospective, deidentified data was obtained from the CUREd Research database hosted by the CURE Group, University of Sheffield. Data was provided under a data sharing agreement which prohibits onward sharing. Other researchers may make their own requests for CUREd data via https://www.sheffield.ac.uk/scharr/research/centres/cure/projects/cured-how-access-data.
