# Supplementary figures and images for: Patient compliance with NHS 111 advice: Analysis of adult call and ED attendance data 2013–2017
Source: PLoS One. 2021 May 10;16(5):e0251362. doi: 10.1371/journal.pone.0251362 (PMC8109810; doi:10.1371/journal.pone.0251362)

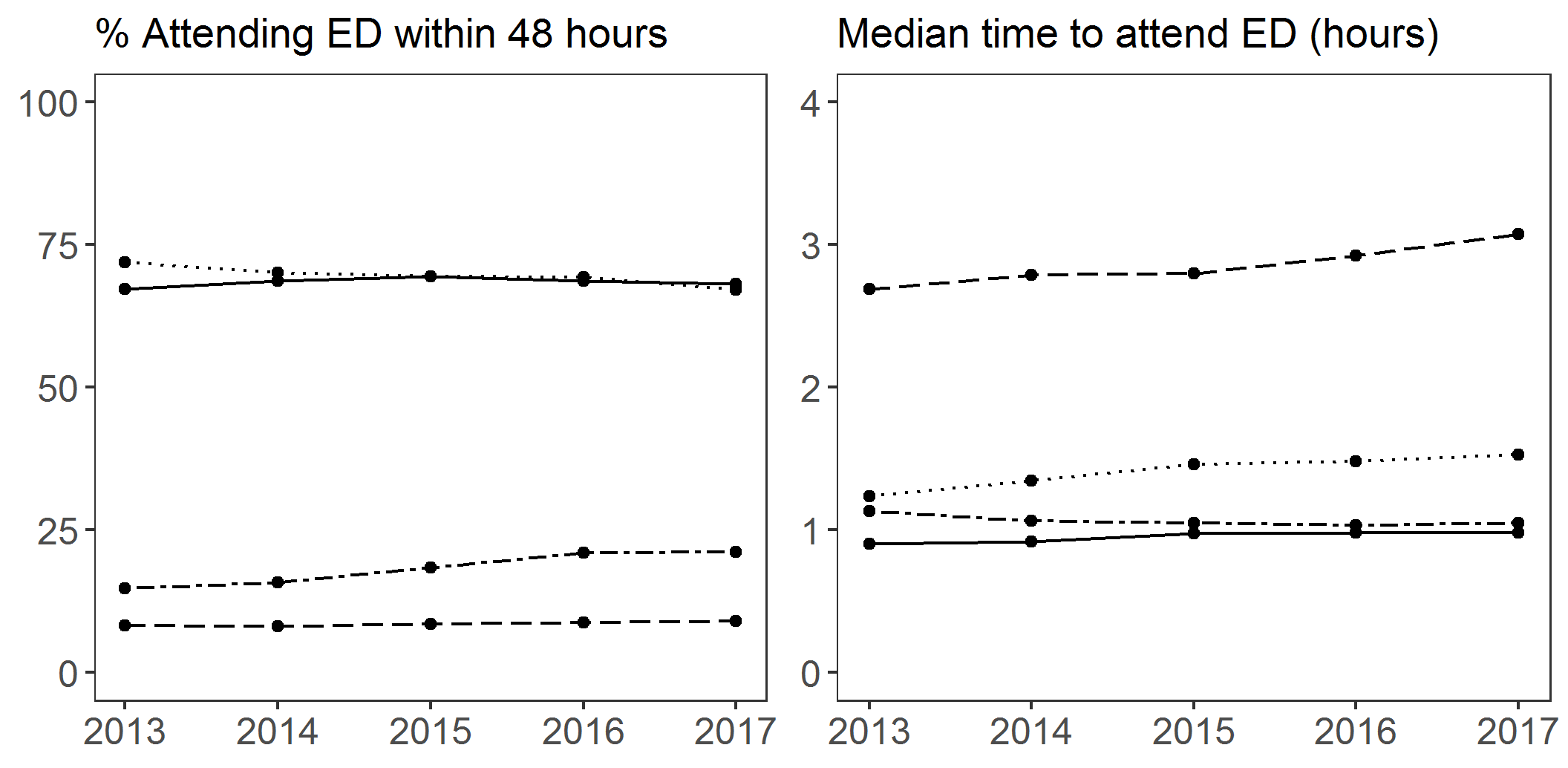

Supplement: S1 Fig — (Solid line = advised to attend ED; dotted line = ambulance dispatched; dashed line = directed to primary care; dot-dash line = advised to self-care). (PNG) [file pone.0251362.s001.png]

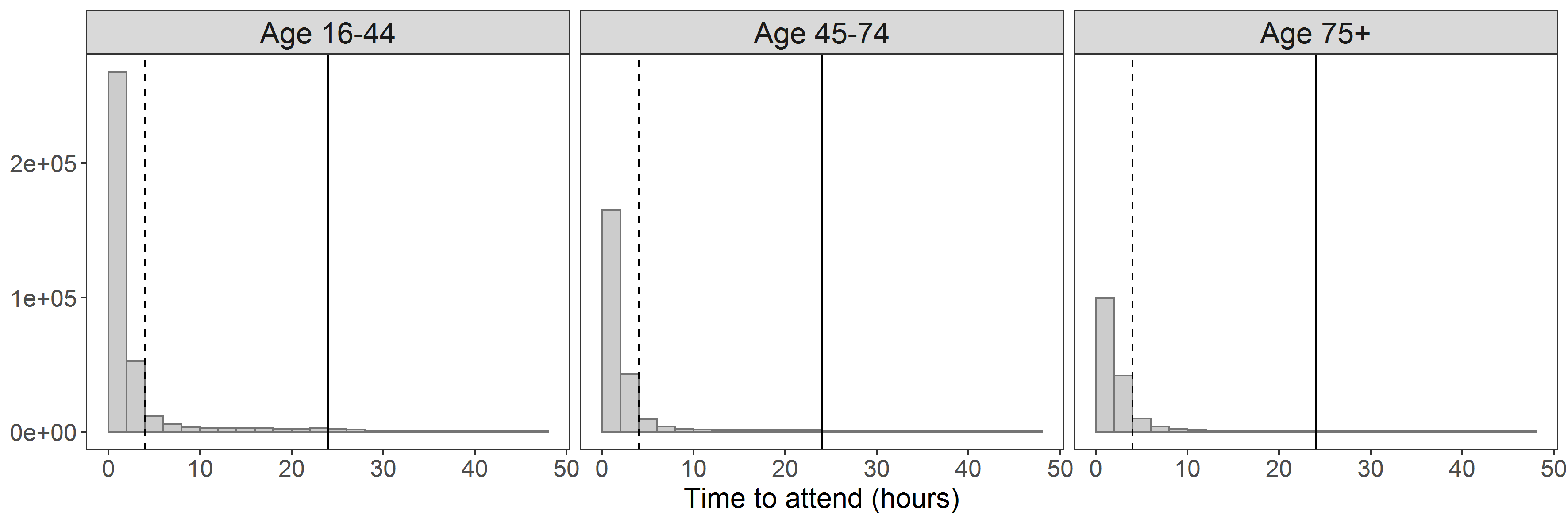

Supplement: S2 Fig — (PNG) [file pone.0251362.s002.png]

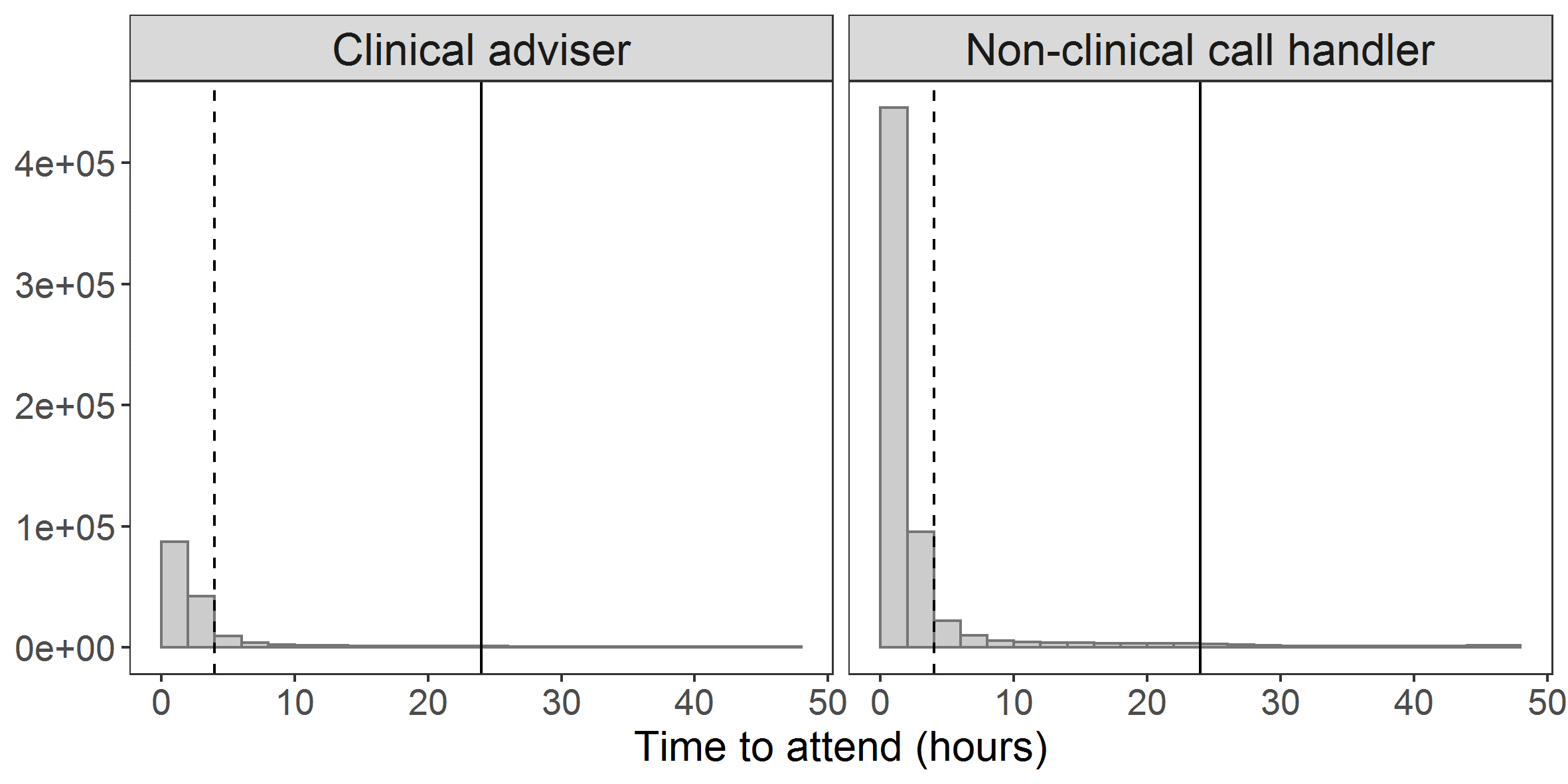

Supplement: S3 Fig — (PNG) [file pone.0251362.s003.png]
